# How many patients are required to provide a high level of reliability in the Japanese version of the CARE Measure? A secondary analysis

**DOI:** 10.1186/s12875-018-0826-2

**Published:** 2018-08-16

**Authors:** Takaharu Matsuhisa, Noriyuki Takahashi, Muneyoshi Aomatsu, Kunihiko Takahashi, Jo Nishino, Nobutaro Ban, Stewart W. Mercer

**Affiliations:** 1 Department of General Medicine/Family & Community Medicine Nagoya University Graduate School of Medicine, 65 Turumai-cho, Showa-ku, Nagoya, 466-8560 Japan; 2 Department of Education for Community-Oriented Medicine, Nagoya University Graduate School of Medicine, 65 Turumai-cho, Showa-ku, Nagoya, Japan; 3 Department of Medical Education, Saku Central Hospital, 197 Usuda, Saku, Japan; 4 Department of Biostatistics Nagoya University Graduate School of Medicine, 65 Turumai-cho, Showa-ku, Nagoya, Japan; 5 Medical Education Center, Aichi Medical University School of Medicine, 1-1 Yazakokarimata, Nagakute, Japan; 6 Department of Primary Care Research in General Practice and Primary Care, Institute for Health and Wellbeing, University of Glasgow, 1 Horselethill Road, Glasgow, G12 9LX Scotland

**Keywords:** Empathy, Consultation, Doctor-patient relationship, General practice, Primary care, Quality of care

## Abstract

**Background:**

Empathy is widely regarded as being key to effective consultation in general practice. The Consultation and Relational Empathy (CARE) Measure is a widely used and well-validated patient-rated measure in English. A Japanese version of the CARE Measure has undergone preliminary validation, but its ability to differentiate between individual doctors has not been established. The current study sought to investigate the reliability of the Japanese version of the CARE Measure in terms of discrimination between doctors.

**Methods:**

We conducted secondary analysis of a dataset involving 252 patients assessed by nine attending General Practitioners. The intra-cluster correlation coefficient was evaluated as an index of the reliability of the Japanese version of the CARE Measure for discriminating between doctors. With a criterion of intra-cluster correlation coefficient = 0.8, we conducted a decision (D) study using generalizability theory to determine the required number of patients for reliable CARE Measure estimates.

**Results:**

The ability of the CARE Measure to discriminate between doctors increased with the number of patients assessed per doctor. A sample size of 38 or more patients provided an average intra-cluster correlation coefficient of 0.8.

**Conclusions:**

The Japanese CARE Measure appears to reliably discriminate between doctors with a feasible number of patient-ratings per doctor. Further studies involving larger numbers of doctors with a multicenter analysis are required to confirm the results of the current study, which was conducted at a single institution.

## Background

Empathy is regarded as a core aspect of effective consultations in general practice [1]. In the context of patient care, Hojat et al. proposes that empathy is primarily a cognitive attribute, not an affective or emotional one. Thus, for a doctor, empathy requires understanding of patients’ experience, concerns and perspectives, as well as the ability to communicate their understanding and their intention to help [2]. Mercer and Reynolds defined empathy in the clinical context as an ability to (i) understand the patient’s situation, perspective, and feelings (and their corresponding meanings), (ii) to communicate that understanding and check its accuracy, and (iii) to act on that understanding with the patient in a helpful and therapeutic way [1]. Empathy has been linked to a number of benefits in health-care encounters, including improved patient satisfaction, better medication adherence, higher patient enablement, and better clinical outcomes [3–6].

Although several tools have been developed to assess physicians’ empathy using self-reported or observer-reported measures [7–9], these methods are limited by doctors’ conceptual structures of empathy, which change with their experiences [10]. Thus, it is ultimately the patient’s perception of empathy that determines the interpersonal effectiveness of the clinical encounter [11]. The Consultation and Relational Empathy (CARE) Measure is a widely used patient-reported measure that has been extensively validated [12]. The CARE Measure was originally developed in English, in the United Kingdom (UK) [13, 14]. It has been translated and validated in other languages, and is currently used by researchers in various countries, including China, Holland, Sweden and Croatia [15–18]. A preliminary study of the validity and internal reliability of a Japanese version of the CARE Measure has been published [19]. However, unlike the English and Chinese versions, the ability of the Japanese version of the measure to effectively discriminate between individual doctors has not yet been established [14, 20, 21].

The current study sought to determine whether the Japanese version of the CARE Measure can reliably differentiate between doctors, and how many patients are required per doctor to provide a high level of reliability.

## Methods

### Data

We conducted a secondary analysis of data from a previous study of the Japanese CARE Measure [19]. We summarize these data below; the full details are given in the original paper [19]. The original data collection using the CARE Measure questionnaire was carried out at the outpatient clinic of General Medicine in the University Hospital in Nagoya, Japan in 2011. Consecutive patients of nine doctors participated, completing a questionnaire in the reception area of the outpatient clinic directly after the consultation. The number of years of experience of the nine doctors ranged from 6 to 33 years. All doctors were male and worked at the same university hospital. All doctors were working as general practitioners (GPs). Three of the doctors were residents, two were teaching staff and four were faculty members. None of the doctors were certified specialist physicians because Family Practitioner certification in Japan only began in 2009. When a doctor felt that asking the participation of a patient might affect their condition (e.g., patients with anxiety disorder) and in cases where patients were unable to answer appropriately because of their disease (e.g., dementia), patients were excluded. Data were collected from July to December of 2011. A total of 252 patients who consulted the nine doctors completed the CARE Measure questionnaire during the study period and were included in the final analysis.

### Data analysis

We evaluated intra-cluster correlation coefficients (ICCs) as a reference index of the reliability of the Japanese CARE Measure, in accord with a previous study [14]. The ICC was defined as:

$$ \mathrm{ICC}=\frac{\sigma_{GP}^2}{\sigma_{GP}^2\kern0.5em +\kern0.5em {\sigma}_P^2\ } $$

where ${\sigma}_{GP}^2$ was the variance in mean CARE Measure score between attending GPs, and ${\sigma}_P^2$ is the variance due to random variation between samples of patients. If the sample size of patients is n, then:

$$ {\sigma}_P^2=\frac{\sigma^2}{n} $$

where *σ*^2^ is the variance of CARE Measure scores between individual patients. We first conducted a *generalizability (G) study* using generalizability theory [22]; ${\sigma}_{GP}^2$ and *σ*^2^ in the ICC (equivalent to a *G-coefficient* in the generalizability theory) were estimated ${\upsigma}_{\mathrm{GP}}^2$using analysis of variance (ANOVA) using G-string IV software [22, 23]. In this analysis, the doctor was considered the object of measurement, and raters (patients) were nested within doctor. We then conducted a *decision (D) study* using generalizability theory [22], in which we determined the number of patients required to achieve the reliability criterion of average ICC = 0.80, as in previous studies of the CARE Measure [20, 24].

## Results

### Patient characteristics across GPs

A total of 252 patients took part in the study. Table 1 shows the characteristics of the patients and the mean CARE Measure scores for each GP. The number of patients participating per doctor ranged from nine to 50. The average number of patients was 28, which was a smaller sample of patients with higher variability than that reported in previous studies of the CARE Measure [14, 20]. There were significant differences in the age of patients between GPs, determined using one-way analysis of variance (ANOVA; *p* < 0.0001). However, there was no significant correlation between CARE Measure scores and age (Spearman’s rank correlation coefficient = 0.001; *P* = 0.990). Therefore, the difference in age among GPs was not considered in the subsequent analysis. There were no significant differences in patients’ gender between GPs, determined using a chi-square test (*p* = 0.963). Mean CARE Measure scores ranged from 34.8 to 45.2. An average score of all patients was 38.8.

**Table 1:** Demographic data of participating patients and outcomes for each GP

	Sample size	Mean age of participants (standard deviation)	Proportion of female participants (%)	Mean CARE Measure score (standard deviation)
GP1	14	42.5 (21.0)	8 (57.1)	34.8 (6.9)
GP2	32	62.8 (12.5)	20 (62.5)	35.3 (8.6)
GP3	15	45.1 (15.0)	8 (53.3)	36.7 (8.0)
GP4	24	59.3 (15.2)	15 (62.5)	37.0 (9.4)
GP5	18	64.5 (14.3)	12 (66.7)	37.7 (6.9)
GP6	47	56.1 (14.7)	33 (70.2)	38.4 (9.1)
GP7	43	52.9 (14.8)	25 (58.1)	39.2 (8.4)
GP8	50	53.5 (15.6)	32 (64.0)	42.9 (7.1)
GP9	9	62.7 (17.6)	6 (66.7)	45.2 (3.8)
All Pt	252	55.6 (16.0)	159 (63.1)	38.8 (8.5)

*Abbreviation*: *GP* general practitioner, *Pt* Patient

### Data analysis

A random effects model implemented in G-string IV software gave ${\sigma}_{GP}^2$ = 6.942 and *σ*^2^ = 66.030. The raters (patients) nested within doctor accounted for most of the variance of the CARE Measure scores (90.5%). Results from a D-study are shown in Table 2. The results indicated that the measure effectively differentiated between doctors with a high degree of reliability with 38 or more patient ratings per doctor (average ICC > 0.8) (Table 2).

**Table 2:** Reliability of Japanese version of the Consultation and Relational Empathy Measure for differentiating between doctors

Number of patients per general practitioner	ICC
1	0.10
10	0.51
20	0.68
30	0.76
35	0.79
38	0.80
40	0.81
50	0.84

*Abbreviation*: *ICC* intra-cluster correlation coefficient

The current data involved a high degree of variability in the number of patients per GP. Thus, we analyzed GPs excluding those who were rated by less than 20 patients. The results revealed that ${\sigma}_{GP}^2$ = 6.365 and *σ*^2^ = 71.298. The ICC was 0.78 with 38 patient reviews per GP, suggesting that 38 patient reviews was an appropriate number.

### Interpretation of individual GPs’ mean score

**Fig. 1:**
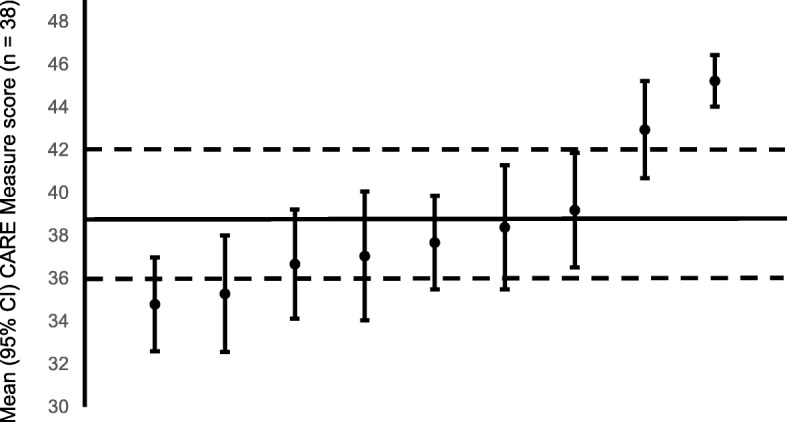
GPs in order of mean CARE Measure score**.** GP mean CARE Measure scores with 95% confidence intervals based on the observed within-GP variance, supposing the ICC was 0.8 (each GP was reviewed by 38 patients). The average score among GPs is shown by the solid line. The dashed lines indicate that GPs with mean scores above 42 or below 36 with a sample of 38 patients have mean scores that are significantly above or below average, respectively

Figure 1 indicates the GPs’ mean CARE Measure scores with 95% confidence intervals based on the observed within-GP variance, supposing the ICC was 0.8 (reviewed by 38 patients per GP). In the present study, the average mean CARE Measure score of nine GPs was 38.6, with a standard deviation of 3.2.

Two GPs scored < 36, five scored 36–42, and two scored > 42. Thus, we used the top and bottom 25% of the distribution to define the cutoffs of 36 and 42 (Fig. 1).

## Discussion

We conducted ICC analysis of data from the Japanese CARE Measure to examine its ability to discriminate effectively between doctors. The current results suggest that the Japanese CARE Measure can effectively differentiate between doctors with 38 or more patient ratings per doctor (average ICC > 0.8). These findings suggest that the measure is feasible for use in routine practice.

Our findings are in accord with previous studies of the reliability of the CARE Measure in languages other than Japanese. A study of the Chinese version of the CARE Measure reported that an average reliability of 0.8 of GPs was achieved with approximately 30 patients per doctor [20]. Similarly, a study of the original English version of the measure tested on GPs in Scotland reported that, for the GP requiring the largest number of patients among attending GPs, 50 patients per doctor resulted in a reliability above 0.8 [14]. We applied the same analysis method to the current data, revealing that the largest patient number required by any GP in our sample was 53, similar to the results of the previous study in Scotland [14].

The heterogeneity of the Chinese version of the mean CARE Measure of GPs was higher (mean score: 34.58; standard deviation: 4.861 in the Chinese version) [20], whereas the heterogeneity in the current study was lower (mean score: 38.6; standard deviation: 3.2). This difference in the required number of patient ratings is likely to be related to studies examining doctors at different stages of training in general practice, resulting in greater variation between doctors. The current study only included GPs who were trained in the same hospital. Thus, the variation between doctors would be expected to be more aligned with the UK study [14] than the Chinese study [20].

A key strength of the current study is its contribution to the development of the Japanese version of the CARE Measure and its future utility. However, the study involved several limitations that should be considered. First, for pragmatic reasons, patients were recruited on a consecutive basis rather than randomly selected. The selection of suitable patients was determined by the attending physician, which may have introduced sample bias. In addition, patients with specific diseases (e.g., anxiety, dementia) were excluded from the study. Because the study was conducted in a single setting, the feasibility of carrying out such research in other settings, such as rural or private clinics, was not tested. The setting used may have been atypical in terms of consultation length and continuity of care. Finally, only nine doctors at the same hospital took part in this study, which was a smaller sample of doctors than in previous studies of the CARE Measure [14, 20].

In our analysis, we chose the outpatient clinic of the university hospital because it provides a primary care facility run by qualified and experienced GPs. GP certification in Japan only began in 2009 and few well-qualified GPs existed in 2011 when the data in the current study were obtained [25]. However, the number of GPs in Japan has increased rapidly since then. Thus, further large multicenter studies including both GPs and non-GPs working as family doctors in Japan would provide valuable insight.

Based on the current results, we believe that the Japanese version of the CARE Measure is useful for evaluating GPs in terms of relational empathy in Japan. Our findings suggest that the Measure is feasible, even within busy clinics. As Japan develops and grows its general practice workforce, ensuring that empathic, patient-centered care is at the heart of the system will aid the acceptability of care for patients, and its future sustainability.

## Conclusion

We validated the reliability of the Japanese version of the CARE Measure in differentiating between doctors. The Measure provides a reliable estimate of perceived GP empathy, if 38 or more completed questionnaires are included. Further comprehensive investigations with larger samples would be valuable for confirming and extending these findings.

## Abbreviations

ANOVA: Analysis of variance
CARE: Consultation and Relational Empathy
GP: General practitioner
ICC: Intra-cluster correlation coefficient
Pt: Patient
UK: The United Kingdom of Great Britain and Northern Ireland
